# Early onset of treatment effects with oral risperidone

**DOI:** 10.1186/1471-244X-7-4

**Published:** 2007-01-19

**Authors:** Thomas J Raedler, Andreas Schreiner, Dieter Naber, Klaus Wiedemann

**Affiliations:** 1 Department of Psychiatry, University Medical Center Hamburg-Eppendorf, Martinistr. 52, 20246 Hamburg, Germany; 2 Department of Medical & Scientific Affairs, Janssen-Cilag, Raiffeisenstr. 8, 41470 Neuss, Germany

## Abstract

**Background:**

The dogma of a delayed onset of antipsychotic treatment effects has been maintained over the past decades. However, recent studies have challenged this concept. We therefore performed an analysis of the onset of antipsychotic treatment effects in a sample of acutely decompensated patients with schizophrenia.

**Methods:**

In this observational study, 48 inpatients with acutely decompensated schizophrenia were offered antipsychotic treatment with oral risperidone. PANSS-ratings were obtained on day 0, day 1, day 3, day 7 and day 14.

**Results:**

Significant effects of treatment were already present on day 1 and continued throughout the study. The PANSS positive subscore and the PANSS total score improved significantly more than the PANSS negative subscore.

**Conclusion:**

Our results are consistent with the growing number of studies suggesting an early onset of antipsychotic treatment effects. However, non-pharmacological effects of treatment also need to be taken into consideration.

## Background

Since their introduction half a century ago, antipsychotics have constituted the backbone of pharmacological treatment of psychosis. In accordance with other groups of psychotropic agents (e.g. antidepressants), a substantial time-lag has been postulated between the initiation of antipsychotic treatment and the onset of the antipsychotic effect. However, this concept of a delayed onset of action has been challenged recently and an earlier onset of antipsychotic treatment effects has been proposed [1,2].

Our group has recently shown that the atypical antipsychotic risperidone is effective in the treatment of acutely decompensated patients with schizophrenia [3]. In this manuscript we present an analysis of the time-course of treatment effects in acutely decompensated patients with schizophrenia, who remained in treatment.

## Methods

A detailed description of the study-design was previously published [3]. In brief, acutely decompensated subjects with schizophrenia were offered treatment with oral risperidone (daily dose 4 – 8 mg given in two doses) upon admission to the secure unit of the Dept. of Psychiatry of the University Medical Center Hamburg-Eppendorf. This unit serves a catchment-area of approximately 250.000 inhabitants of the city of Hamburg, Germany. Benzodiazepines (diazepam 5 – 60 mg/d and lorazepam 0.5 – 10 mg/d) as well as anticholinergics (biperiden 2 – 8 mg/d) were added as clinically indicated. The demographic information of the treatment-group is listed in Table 1.

**Table 1:** Demographic information

Number of participants:	48 (25 males/23 females)
Age:	36.9 ± 13.4 years (range 18 – 68 years)
PANSS at admission:	110.9 ± 20.4 (range 72 – 158)
CGI at admission:	5.9 ± 0.6 (range 5 – 7)
Duration of treatment:	13.4 ± 9.7 days (range 1 – 31 days)
Maximum dose of risperidone:	6.1 ± 1.4 mg/d (range 4 – 8 mg/d)

Subjects were rated with the PANSS and CGI on day 0 (baseline), day 1, day 3, day 7 and day 14. For the PANSS, we analysed the total score as well as the positive, negative and general psychopathology scales. Due to weekends and holidays, four subjects were not rated on day 1. All other ratings were performed as scheduled. For easier comparison, all ratings were expressed as percentage of the baseline-values (day 0 = 100%).

### Statistical Analysis

We performed an observed cases analysis for the first two weeks of treatment. Wilcoxon's matched pairs test was used for comparison of symptom-ratings with baseline-ratings. Friedman's two-way analysis of variance was used to assess statistical differences between the different PANSS-subscores for each day.

## Results

48 subjects participated in this open-label observational study. Subjects were diagnosed with schizophrenia (n = 40), schizoaffective disorder (n = 5) or schizophreniform disorder (n = 3) according to DSM-IV-TR.

Data are available for 43 patients on day 1 (89.6%), 37 patients on day 3 (77.1%), 32 patients on day 7 (66.7%) and 25 patients on day 14 (52.1%). The reasons for discontinuation of risperidone are shown in Table 2.

**Table 2:** Reasons for discontinuation of risperidone

	**n**
Discharge against medical advice	7
Regular discharge	2
Intramuscular injection	8
Switch to another antipsychotic	4
**Noncompliance**	**2**

All PANSS ratings showed a significant improvement with antipsychotic treatment. Table 3 lists the PANSS total scores as well as the different PANSS subscores for each time-point.

**Table 3:** Total PANSS-scores and PANSS-subscores. For easier comparison, ratings on day 1, day 3, day 7 and day 14 are expressed as percentage of base-line (day 0 = 100%)

	**PANSS Total**	**PANSS Positive**	**PANSS Negative**	**PANSS Gen. Psychopath.**
**Day 0 **(n = 48)	100%	100%	100%	100%
**Mean ± SD**	110.9 ± 20.4	30.4 ± 5.2	25.3 ± 7.3	55.1 ± 11.8
**Day 1 **(n = 43)	96.7% ± 6.7%	97.7% ± 9.3%	96.8% ± 9.7%	96.3% ± 6.7%
**Day 3 **(n = 37)	87.3% ± 12.9%	85.1% ± 14.3%	91.1% ± 19.5%	88.0% ± 14.3%
**Day 7 **(n = 32)	76.6% ± 17.5%	69.6% ± 21.8%	83.6% ± 25.8%	79.0% ± 17.5%
**Day 14 **(n = 25)	68.0% ± 19.0%	62.6% ± 25.2%	72.4% ± 19.6%	69.9% ± 18.1%

On day 1, the PANSS total score as well as the PANSS negative subscale and the PANSS general psychopathology subscale decreased significantly compared to baseline (n = 43; all Z > 2.92; all p < 0.01). The positive symptoms subscale of the PANSS on day 1 showed no significant difference when compared to baseline (n = 43; Z = 1.46; p < 0.15).

On day 3, the positive symptom subscale of the PANSS was significantly lower than the baseline-rating. The PANSS total score as well the PANSS negative subscale and the PANSS general psychopathology subscale also decreased further. All scores were significantly lower than the baseline-scores on day 3 (n = 37; all Z > 3.61; all p < 0.001). On day 7 and day 14, a highly significant decrease in the ratings of total PANSS and all PANSS subscales was observed compared to baseline (all Z > 3.29; all p < 0.001).

Comparing the changes between the subscales of the PANSS, significant differences were found on day 7 (X^2 ^= 23.7; p < 0.001) and day 14 (X^2 ^= 15.1; p < 0.01). While all ratings improved, the decrease in symptom severity was most pronounced on the PANSS positive subscale. In contrast, the ratings on the PANSS negative subscale showed the smallest decrease over time. Further analysis revealed that the decrease in symptom rating was significantly higher on the positive subscale (Z = 3.80; p < 0.001) and the PANSS total score (Z = 2.59; p < 0.01) than on the PANSS negative subscale on day 7. Similar differences were found on day 14 with a significantly stronger improvement in the PANSS positive subscore (Z = 2.62; p < 0.01) and a trend towards significance for the PANSS total score (Z = 1.92; p = 0.05) compared to the PANSS negative subscale.

## Discussion

Over the past decades, the concept of a delayed onset of antipsychotic effects on symptoms of acute schizophrenia has been generally accepted. This concept included the understanding that first effects after the initiation of antipsychotic treatment were not seen until after several weeks of treatment.

The dogma of a delayed onset of action has only recently been challenged for antipsychotic treatment [1,2] as well as for antidepressant treatment [4,5]. Our data provide additional support for the concept of an early onset of action of antipsychotic treatment in acutely decompensated patients with schizophrenia. Our results suggest that in severely ill subjects who remained in treatment, first effects of treatment can be demonstrated as early as after one day of treatment. Over the course of the next two weeks, symptom ratings for positive, negative and general symptoms of schizophrenia decreased further. While positive symptoms responded even better to antipsychotic treatment, we still saw a significant improvement in negative symptoms during the first two weeks of treatment.

The observational nature of this study without a placebo-group precludes definite statements on the time-course of antipsychotic treatment effects. Still, our data are consistent with previously published studies. Looking at the effects of intramuscular medications, both intramuscular haloperidol and olanzapine led to an improvement in symptoms of schizophrenia within the first 24 hours of treatment [6]. In a large meta-analysis of antipsychotic response, a decrease in psychotic symptom ratings (e.g. PANSS and BPRS) of 13.8% was seen during the first week of treatment. The second week of treatment led to an additional decrease in symptom-ratings of 8.1% [1]. Similar acute treatment effects were seen with the atypical antipsychotic amisulpride with the largest improvement in psychotic symptoms occurring within the first two weeks of treatment [2]. Both studies found a larger symptom reduction during the first two weeks of treatment than during consecutive weeks. An onset of action during the first week of treatment across a broad range of symptoms was also described for the second generation antipsychotic quetiapine [7]. The atypical antipsychotic risperidone has been associated with an earlier onset of action than haloperidol [8], clozapine [9] and zuclopenthixol [10] in the treatment of schizophrenia. While the rate of improvement of symptoms in our study exceeds some of these studies [e.g. [1,2]], it should be kept in mind that subjects who discontinued treatment were excluded from further analysis.

Potential effects of non-pharmacological interventions should be taken into consideration when assessing the results of this study. We studied a group of patients with schizophrenia who were admitted to a secure unit with acute psychotic decompensation. All subjects were treated with oral risperidone in a daily dose between 4 and 8 mg as well as benzodiazepines as needed. In addition to the pharmacological intervention, all subjects received skilled nursing-care in the setting of an acute treatment ward. Non-pharmacological interventions included a safe, structured and quiet environment, the normalization of food- and fluid-intake as well as adequate opportunity to rest. Concomitant alcohol- and substance-use, which may have contributed to the exacerbation of psychosis, also came to an end with the admission. In addition, precipitating events were addressed during the in-patient treatment. These non-pharmacological factors need to be taken into consideration when assessing the effects of acute treatment. However, in clinical practice, a substantial number of patients with schizophrenia refuse antipsychotic treatment and fail to stabilize despite being placed in a quiet and safe environment. The lack of improvement in patients who refuse antipsychotic treatment points to a specific role of the pharmacological treatment in the stabilization of acutely decompensated patients with schizophrenia.

## Conclusion

Our data add to the growing number of studies showing an early onset of antipsychotic effects in subjects with acutely exacerbated schizophrenia. While full benefits of antipsychotic treatment may take several weeks to months, first effects of antipsychotic treatment can be seen as early as after one day of treatment. However, non-pharmacological effects also need to be taken into consideration.

## List of abbreviations

BPRS: Brief Psychiatric Rating Scale

CGI: Clinical Global Impression scale

PANSS: Positive and Negative Syndrome Scale

## Competing interests

Dr. Raedler, Prof. Naber and Prof. Wiedemann have received honoraria, grant-support and/or travel funds from Janssen Pharmaceuticals. Dr. Schreiner is an employee of Janssen-Cilag Germany.

## Authors' contributions

TJR conceived of the study, participated in the design and coordination and drafted the manuscript.

AS participated in the design and coordination, helped with the statistical analysis and drafted the manuscript

DN participated in the design and coordination of the study

KW conceived of the study and participated in the design and coordination.

All authors read and approved the final manuscript

## Pre-publication history

The pre-publication history for this paper can be accessed here:


